# Unravelling Contributions of Astrocytic Connexin 43 to the Functional Activity of Brain Neuron–Glial Networks under Hypoxic State In Vitro

**DOI:** 10.3390/membranes12100948

**Published:** 2022-09-28

**Authors:** Tatiana A. Mishchenko, Roman S. Yarkov, Mariia O. Saviuk, Mikhail I. Krivonosov, Alexey D. Perenkov, Sergey V. Gudkov, Maria V. Vedunova

**Affiliations:** 1 Prokhorov General Physics Institute of the Russian Academy of Sciences, 119991 Moscow, Russia; 2 Institute of Biology and Biomedicine, Lobachevsky State University of Nizhny Novgorod, 23 Gagarin Ave., 603022 Nizhny Novgorod, Russia; 3 Institute of Information, Technology, Mathematics and Mechanics, Lobachevsky State University of Nizhny Novgorod, 23 Gagarin Ave., 603022 Nizhny Novgorod, Russia

**Keywords:** gap junctions, Cx43, Gap19, hypoxia, primary cortical cultures, functional network activity, calcium imaging

## Abstract

Brain hypoxia remains an Achilles’ heel for public health that must be urgently addressed. Hypoxic damage affects both neurons and glial cells, particularly astrocytes, which are in close dynamic bi-directional communication, and are organized in plastic and tightly regulated networks. However, astroglial networks have received limited attention regarding their influence on the adaptive functional rearrangements of neural networks to oxygen deficiency. Herein, against the background of astrocytic Cx43 gap junction blockade by the selective blocker Gap19, we evaluated the features of spontaneous calcium activity and network characteristics of cells in primary cultures of the cerebral cortex, as well as the expression levels of metabotropic glutamate receptors 2 (mGluR2) and 5 (mGluR5) in the early and late periods after simulated hypoxia in vitro. We showed that, under normoxic conditions, blockade of Cx43 leads to an increase in the expression of metabotropic glutamate receptors mGluR2 and mGluR5 and long-term modulation of spontaneous calcium activity in primary cortical cultures, primarily expressed in the restructuring of the functional architectonics of neuron–glial networks through reducing the level of correlation between cells in the network and the percentage of existing correlated connections between cells. Blocking Cx43 during hypoxic injury has a pronounced neuroprotective effect. Together with the increased expression of mGluR5 receptors, a decrease in mGluR2 expression to the physiological level was found, which suggests the triggering of alternative molecular mechanisms of cell adaptation to hypoxia. Importantly, the blockade of Cx43 in hypoxic damage contributed to the maintenance of both the main parameters of the spontaneous calcium activity of primary cortical cultures and the functional architectonics of neuron–glial networks while maintaining the profile of calcium oscillations and calcium signal communications between cells at a highly correlated level. Our results demonstrate the crucial importance of astrocytic networks in functional brain adaptation to hypoxic damage and could be a promising target for the development of rational anti-hypoxic therapy.

## 1. Introduction

Over the last decade, the problem of the emergence and development of brain hypoxia remains one of the key issues that must be urgently addressed. The brain is an Achilles’ heel for hypoxic damage due to the need for high oxygen consumption to carry out its functioning and to maintain viability as well as a limited range of antioxidant enzymes and compensatory capacity [1,2,3,4,5]. The launch of hypoxia-induced pathological mechanisms not only leads to cell death and significant alterations in brain functioning, but also accompanies the development of severe pathologies of the central nervous system (CNS), including ischemic stroke, a number of neurodegenerative diseases and brain cancer [6,7,8,9,10].

There is no doubt that hypoxic damage affects both neurons and glial cells [11,12,13]; however, glial cells have received only limited attention with regard to the brain’s adaptation to oxygen deficiency. Glial cells, particularly astrocytes, and neurons are in close dynamic bi-directional communication, and both are organized in plastic and tightly regulated networks [14,15]. Astrocytes form astroglial networks through gap junctions capable of regulating the exchange between the cytoplasm and the extracellular space and processing signaling activity [14,15,16]. The gap junction structure is formed by the interaction of connexons, so-called hemichannels, on adjacent cells. One connexon is assembled by six connexin protein molecules, among which connexin 43 (Cx43) and connexin 30 (Cx30) mainly provide communication between astrocytes in the brain [14,15,17]. Astrocytic gap junctions provide the rapid intercellular exchange of ions and metabolites, which is crucial for buffering K^+^ and glutamate ions, Ca^2+^ maintenance and the propagation of calcium waves and synaptic plasticity at the network level [15,18,19,20]. Deficiency of Cx43 protein leads to a decrease in the number of gap junctions, resulting in the disruption of calcium wave propagation, which significantly modulates the neural networks’ activity [17].

Studies of the last decade have shown that astrocyte Cx43 is critically involved in promoting hypoxia–ischemia injury. Cx43, forming the gap junctions and hemichannels, is essential for the trafficking of glutamate, levels of which are highly elevated upon hypoxia–ischemia, and the development of excitotoxicity and neural cell death [21]. Cx43 protein levels increase within the first six hours after ischemia modeling in the cortical tissue and it remains detectable in the peri-infarct zone for the following four days [22]. Upregulation of Cx43 expression can mediate the delayed and prolonged apoptotic-like astrocytic death and the decreased cell viability after the hypoxia–ischemia injury [23]. The blockade or inhibition of astrocyte Cx43 hemichannels after ischemia–reperfusion effectively reduced glutamate levels in the astrocytes and neurons in vitro, suggesting the maintenance of cell viability and the potential for the development of novel neuroprotective therapy [23,24,25]. On the other hand, Cx43 hemichannels can act in a close relationship with inflammation and neurotoxicity by releasing inflammatory factors and glutamate in ischemic states [21,26,27,28,29]. A larger stroke volume and increased apoptotic processes and inflammation were shown after focal brain ischemia in mice lacking Cx43 in astrocytes [30].

Further studies are required to determine whether alterations in Cx43 gap junction channels influence the functional reorganization of neural–glial network interactions following hypoxia. The present study reveals the role of inter-astrocytic interactions in the functional activity of hypoxia-induced neuron–glial networks. Against the background of Cx43 inhibition by the selective blocker Gap19 using the calcium imaging technique and original mathematical analysis algorithms, we evaluated the features of spontaneous calcium activity and network characteristics of cells in primary cultures of the cerebral cortex during acute normobaric hypoxia modeling. To better understand the molecular mechanisms underlying inter-astrocytic and astro-neuronal interaction, we used the RT-qPCR procedure to analyze the expression levels of metabotropic glutamate receptors 2 (mGluR2) and 5 (mGluR5) against the background of the blockade of Cx43 and hypoxic damage.

## 2. Materials and Methods

### 2.1. Ethics Statement

The animals were housed in a certified SPF vivarium at Lobachevsky State University of Nizhny Novgorod. All experimental procedures were approved by the Bioethics Committee of Lobachevsky University (ethic code No 58 from 22 November 2021) and carried out in accordance with Act 708n (23 August 2010) of the Russian Federation National Ministry of Public Health, which states the rules of laboratory practice for the care and use of laboratory animals, and Council Directive 2010/63 EU of the European Parliament (22 September 2010) on the protection of animals used for scientific purposes. Pregnant C57BL/6 mice (day of gestation 18) were sacrificed by cervical vertebra dislocation.

### 2.2. Isolation and Characterization of the Primary Cerebral Cortex Cell Cultures

Primary cortical cells were obtained from the cerebral cortices of C57BL/6 mice embryos (day of gestation 18) and seeded on culture plates pretreated with polyethyleneimine solution (1 mg/mL) (Sigma-Aldrich, Darmstadt, Germany) according to the previously developed protocol [31,32]. After surgical isolation, the tissue of embryonic cerebral cortices was subjected to enzymatic digestion with 0.25% solution of trypsin–ethylenediaminetetraacetic acid (EDTA, Invitrogen, Waltham, MA, USA) for 20 min. The suspension of dissociated cells was centrifuged at 1000 rpm for 3 min and was then seeded on a culture plate at an approximate initial density of 7000–9000 cells/mm^2^. The primary cortical cultures were cultivated in Neurobasal medium (ThermoFisher Scientific, Waltham, MA, USA) supplemented with 2% B27 (Thermo Fisher Scientific, Waltham, MA, USA), 0.5 mM L-glutamine (ThermoFisher Scientific, Waltham, MA, USA) and 0.4% fetal bovine serum (Biosera, Nuaillé, France) under constant conditions of 37 °C, 5% CO_2_ and a humidified atmosphere in a Binder C150 incubator (BINDER GmbH, Tuttlingen, Germany). Half of the medium was replaced once every three days.

In the course of method optimization, we performed immunocytochemical staining on day 14 of primary cortical culture development in vitro (DIV) in order to analyze the cellular content. The fixed primary cortical cultures (4% paraformaldehyde solution containing 4% sucrose, 15 min) were treated with a detergent solution (0.5% Triton X-100, 0.1% Tween 20 and 5% goat serum in PBS) and then incubated with the following primary antibodies within two hours: polyclonal rabbit anti MAP2 (a marker of differentiated neurons) (1:500 dilution, Abcam 32454, Cambridge, UK), a polyclonal chicken anti-GFAP (a marker of differentiated astrocytes) (1:500 dilution, Abcam ab4674, Cambridge, UK). After three steps of washing with 0.1% Triton X-100 and 0.1% Tween 20 in PBS, the cultures were subjected to a two-hour incubation with the secondary antibodies: chicken anti-Rabbit Alexa Fluor^®^ 647 (1:800 dilution, ThermoFisher Scientific, A-21245, Waltham, MA, USA) and goat anti-chicken Alexa Fluor^®^ 555 (1:800 dilution, ThermoFisher Scientific, A-21437, Waltham, MA, USA). The immunostained cultures were embedded in ProLong™ Gold antifade reagent with DAPI (Thermo Fisher Scientific, 2260869, Waltham, MA, USA) and examined using an LSM 800 confocal laser scanning microscope (Zeiss, Oberkochen, Germany).

**Figure 1:**
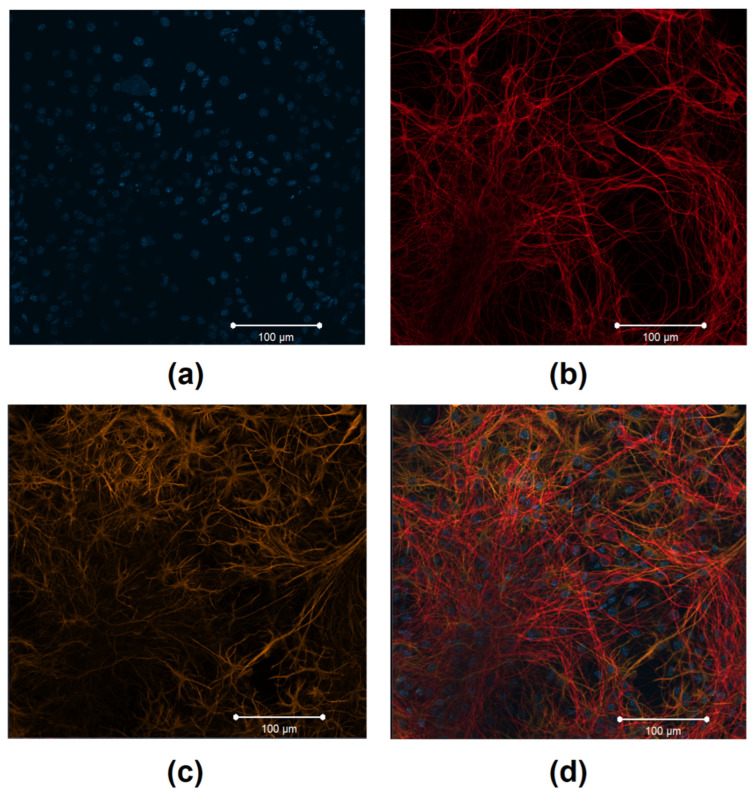
Immunocytochemical analysis of primary cortical cultures on day 14 of cultivation in vitro. Representative confocal images were obtained using an LSM 800 confocal laser scanning microscope (Zeiss, Oberkochen, Germany). (**a**) Fluorescence of a nuclear marker (DAPI) (λex 350 nm; λem 470 nm); (**b**) fluorescence of a marker of neuronal protein (MAP2) (λex 594 nm; λem 650–665 nm); (**c**) fluorescence of a marker of cytoskeleton protein of differentiated astrocytes (GFAP) (λex 488 nm; λem 555–580 nm); (**d**) overlay of the fluorescence channels. Scale bars: 100 μm.

Immunocytochemical analysis revealed that the cellular content of primary cortical cultures on DIV 14 was characterized by the presence of neurons and glial cells in an approximate ratio of 1:2 (Figure 1). These findings are consistent with data on the cellular ratio of primary hippocampal cultures, for which the prevalence of a population of mature chemical synapses with mature axo-dendritic and axo-spiny asymmetric contacts and stable functional activity of neuron–glial networks with a complex characteristic pattern was shown previously [33,34,35].

### 2.3. Experimental Design

The experiments were conducted beginning on day 14 of primary cortical culture development in vitro.

Gap19, a selective connexin 43 inhibitor (Sigma-Aldrich, Darmstadt, Germany), was used to study the contribution of inter-astrocytic contacts to the functional neuron–glial network activity during hypoxic damage.

Acute normobaric hypoxia was modeled on DIV 14, according to the previously developed protocol [32,36], by replacing the culture medium with a medium with a low oxygen content for 10 min. The hypoxic medium was created by passing argon gas through the Neurobasal medium in a sealed chamber at a pressure of 1–1.5 MPa. The control cultures were subjected to total replacement of the culture medium by a complete growth medium with normal oxygen content. Gap19 was added to the culture medium at a concentration of 10 μM 20 min before the hypoxia simulation.

The expression levels of metabotropic receptors mGluR2 and mGluR5 in cells of primary cortical cultures were assessed on days 1 and 3 after hypoxia modeling using the RT-qPCR technique. In the late post-hypoxic period, we registered spontaneous calcium activity and evaluated the network characteristics of primary cortical cultures.

### 2.4. RNA Extraction and RT-qPCR

Quantitative real-time PCR analysis was used to analyze the expression levels of the metabotropic glutamate receptor 2 (mGluR2) and metabotropic glutamate receptor 5 (mGluR5) based on the protocol described in our previous works [32,33]. Total RNA was isolated from primary cortical cultures using an ExtractRNA kit (Evrogen, Moscow, Russia). The synthesis of cDNA was performed using the Moloney murine leukemia virus (MMLV) reverse transcriptase (Evrogen, Moscow, Russia) and specific primers (Oaz1_rv, Grm2 R, Grm5 R).

RT-qPCR reactions were performed using qPCRmix-HS SYBR + LowROX (Evrogen, Moscow, Russia) and a CFX96 Touch Real-Time PCR Detection System (Bio-Rad, Hercules, CA, USA).

The following pairs of primers were used:

Oaz1_fw5′-AAGGACAGTTTTGCAGCTCTCC-3′;

Oaz1_rv5′-TCTGTCCTCACGGTTCTTGGG-3′;

Grm2 F5′–TCCAGTGATTATCGGGTGCA-3′;

Grm2 R5′–AAGCTGGGATCCAGACCCTT-3′;

Grm5 F5′–CTGTGCACACAGAAGGCAAC-3′;

Grm5 R5′–TCAGCCCAGCCATCACTGC-3′.

The results were processed by the ΔΔCt method using a reference sample, in which the level of expression of the target genes was taken as a unit. Normalization was performed relative to the reference gene (Oaz1).

### 2.5. Functional Calcium Imaging

Functional calcium activity of primary cortical cultures was analyzed using the calcium imaging technique. Spontaneous calcium activity of cells was recorded using a calcium-sensitive dye, Oregon Green 488 BAPTA-1 AM (0.4 mM, ThermoFisher Scientific, Waltham, MA, USA), and an LSM 800 confocal laser scanning microscope (Zeiss, Oberkochen, Germany), according to the previously developed protocol [32,37]. The Oregon Green fluorescence was excited at 488 nm with an argon laser, and the emission was recorded in the range of 500–530 nm. The time-series of confocal images at the registration rate of two frames per second were recorded for the analysis of the dynamics of changes in the intracellular calcium concentration. The recording time was 10 min; at least 3 fields of view in each culture were assessed.

The biological noise was calculated relative to fibrosarcoma MCA205 cells, which have a non-neuronal origin and are not characterized by calcium intercellular signaling. Based on the biological noise and confocal microscope noise, the dF/F0 threshold value was set as 0.08. Values exceeding the threshold were identified as calcium events. The following parameters were assessed: percentage of cells exhibiting Ca^2+^ activity (%); the duration (time from the beginning to the end of an oscillation, s) and frequency (average number of oscillations per min) of Ca^2+^ oscillations.

### 2.6. Network Characteristic Assessments in the Cerebral Cortex Cell Cultures

The analysis of the functional state of the neuron–glial networks was based on a previously developed algorithm for the detection of fluctuations in the intracellular calcium level in cells of primary brain cell cultures [38,39]. The algorithm allows the neuron–glial network representation as an oriented graph, the nodes of which correspond to individual cells, and the edges connect the corresponding nodes and indicate a significant correlation between pairs of cells (*p* > 0.3). The spread of calcium signals between cells results in detecting time delays in the increase in Ca^2+^ concentration.

The following parameters were analyzed: the correlation between cells, the average number of connections per cell, the ratio of the available connections in the culture to the maximum possible number of connections in the culture and the signal speed propagation.

### 2.7. Statistical Analysis

Statistical analysis was performed using GraphPad Prism v.9.3.1.471 https://www.graphpad.com/ (accessed on 2 December 2021) (San Diego, CA, USA). RT-qPCR data were expressed as “M [Q1; Q3]”, where M—median, Q1—first quartile (quantile 0.25) and Q3—third quartile (quantile 0.75), and analyzed using the Mann–Whitney test. Calcium imaging data were presented as the mean ± standard error of the mean (SEM) and analyzed using the Kruskal–Wallis nonparametric ANOVA. A difference was considered to be statistically significant if *p* < 0.05. At least three independent biological replicates were used for all experiments.

## 3. Results

### 3.1. Assesment of the Expression Levels of Metabotropic Glutamate Receptors mGluR2 and mGluR5 in Primary Cerebral Cortex Cells under Blockade of Cx43 and Hypoxic Damage

**Figure 2:**
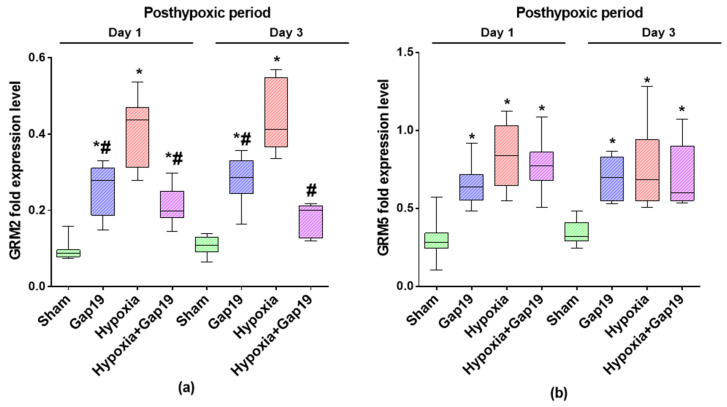
Assessment of the levels of mRNA expression of metabotropic glutamate receptors mGluR2 and mGluR5 in primary cerebral cortex cells in the post-hypoxic period. Data were normalized relative to values of reference gene Oaz1. (**a**) Day 1 after hypoxia exposure; (**b**) Day 3 after hypoxia exposure. * versus “Sham”, # versus “Hypoxia”; *p* < 0.05, the Mann–Whitney test.

Since Cx43 in gap junctions and hemichannels is essential for the trafficking of glutamate [21], whose dysregulation accompanies hypoxic damage, at the first stage of the study, we paid attention to the features of the expression of the key links of the glutamatergic system: metabotropic glutamate receptors 2 (mGluR2) and 5 (mGluR5) (Figure 2).

It was demonstrated that, under normal conditions, blockade of Cx43 promotes the long-term stimulation of mGluR2 receptor expression (Figure 2a). The day after the Gap19 blocker application, the level of mGluR2 expression became significantly higher than the values of the “Sham” group (by an average of 2.7 times). An increased level of mGluR2 expression in the Gap19 group relative to sham values persisted for three days after the blockade of Cx43 (Figure 2a).

Hypoxic damage leads to a pronounced increase in mGluR2 receptor expression. On the first day of the post-hypoxic period, the expression level of mGluR2 in the “Hypoxia“ group exceeded the values of the “Sham” group by an average of 4.4 times and the values of the “Gap19“ group by 1.6 times. On the third day after hypoxia modeling, the expression level of mGluR2 exceeded the values of the “Sham” group and the “Gap19” group by an average of 4.1 and 1.6 times, respectively (Figure 2a).

The blockade of Cx43 during hypoxic exposure contributed to a decrease in the expression levels of mGluR2 receptors relative to the “Hypoxia” group by an average of 1.9 times and a return to the physiological level in the late post-hypoxic period. There were no significant differences between the “Sham” and “Hypoxia+Gap19” groups on day 3 after hypoxia modeling (*p* > 0.05, the Mann–Whitney test) (Figure 2a).

The blockade of Cx43, both in normal conditions and hypoxic injury, led to an increase in the expression levels of mGluR5 receptors (Figure 2b). At the time interval of 24 h, the values in the “Gap19” and “Hypoxia+Gap19” groups exceeded those of the “Sham” group by 2.2 times and 2.6 times, respectively, and remained elevated for the following two days. Similar dynamics were noted in the “Hypoxia” group. The day after hypoxia modeling, the values of the expression levels of mGluR5 receptors in the “Hypoxia” group were on average 2.8-fold higher than those in the “Sham” group; on the third day of the post-hypoxic period, they were higher by 2.2 times.

### 3.2. Features of Spontaneous Calcium Activity of Primary Cerebral Cortex Cell Cultures under Cx43 Blockade and Hypoxic Damage

Brain functionality depends not on individual cells but rather on their functional ensembles, which constitute complex neuron–glial networks. Studies on the functional activity of neuron–glial networks are a key aspect of a comprehensive analysis of ongoing physiological and pathological processes in the CNS, which allows us to evaluate the features of both the complex response of the network and the contribution of its every element in response to stimuli or pathological influences [32,33,34,37]. The calcium imaging technique is a powerful tool for registering the spatiotemporal patterns of neural–glial network metabolic activity, visualization of the architecture and mapping the activity of networks with cellular resolution [33].

**Figure 3:**
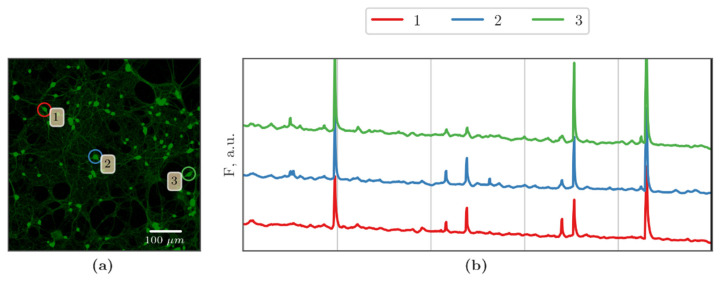

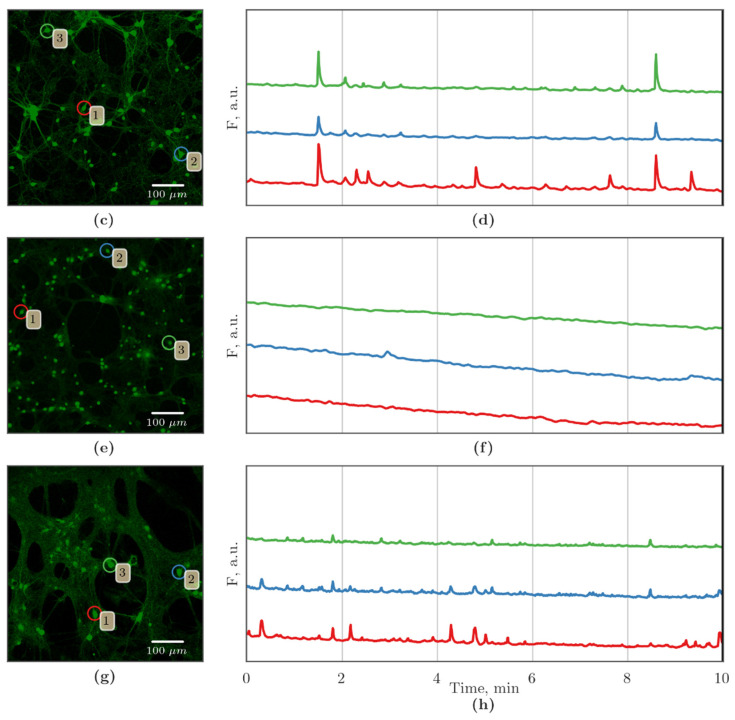
Representative recordings of spontaneous calcium activity of cells in primary cortical cultures on the third day of the post-hypoxic period: (**a**,**b**) Sham; (**c**,**d**) Gap19; (**e**,**f**) Hypoxia; (**g**,**h**) Hypoxia+Gap19. Scale bars: 100 μm.

In this regard, we studied the features of the functional calcium activity of cells in primary cortical cultures in the later period after the blockade of Cx43 and hypoxia modeling (Figure 3, Videos S1–S4 in Supplementary Materials).

**Figure 4:**
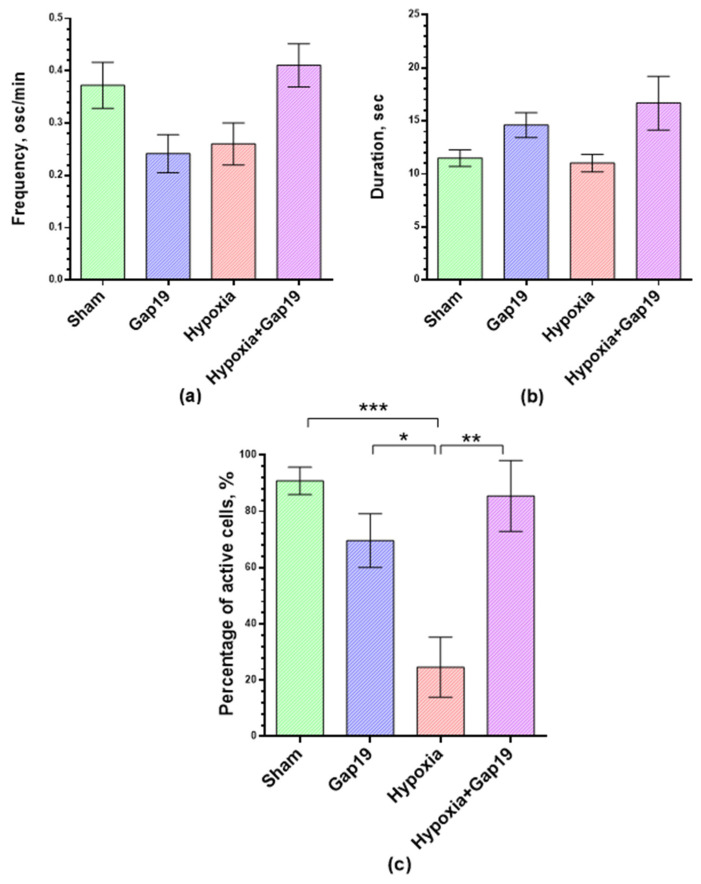
Main parameters of functional calcium activity of primary cortical cultures on the third day of the post-hypoxic period. (**a**) Number of Ca^2+^ oscillations per min; (**b**) duration of Ca^2+^ oscillations; (**c**) proportion of cells exhibiting Ca^2+^ activity. * *p* < 0.05; ** *p* < 0.01, *** *p* < 0.001, Kruskal–Wallis test.

Our analysis did not reveal any pronounced negative effects of Cx43 blockade on the main parameters of calcium activity in primary cortical cells under normoxic conditions (Figure 3b and Figure 4). Nevertheless, the cultures of the “Gap19” group showed a trend towards a decrease in the percentage of functionally active cells (69.57 ± 9.53%) relative to the sham values (90.79 ± 4.87%). Ca^2+^ oscillations have, on average, a frequency of 0.24 ± 0.04 osc/min and a duration of 4.60 ± 1.17 s that formed a calcium activity profile close to that of the sham cultures (Figure 3 and Figure 4) and corresponded to the values of primary hippocampal cultures in a normal state [32,33,34,35,37,40].

Hypoxia modeling caused significant changes in the functional calcium activity of primary cortical cultures. On day 3 of the post-hypoxic period, the percentage of active cells in the “Hypoxia” group (24.63 ± 10.65%) decreased dramatically relative to the sham values, by more than five times on average (Figure 4c). The blockade of Cx43 has a pronounced neuroprotective effect under hypoxic damage. On the third day of the post-hypoxic period, the high percentage of functionally active cells (85.47 ± 12.62%) and the calcium activity profile remained in the “Hypoxia+Gap19” group at the intact level. Nevertheless, there is a tendency to increase the duration of Ca^2+^ oscillations, which can affect the network characteristics of cells in primary cortical cultures, which we analyzed further.

### 3.3. Features of Neuron–Glial Network Interactions in Primary Cerebral Cortex under Cx43 Blockade Hypoxic Damage

In a normal state, most cells of the neuron–glial network are in dynamic interaction with each other, which determines the synchronized functional activity of the network. The death of functionally important elements in response to a stress factor or a pharmacological agent can lead to network restructuring, leading to its simplification and pronounced functional impairment. The potential death of neuron–glial network elements is evidenced by the lack of correlation in the profile of Ca^2+^ fluctuations between functionally active cells [33,37].

**Figure 5:**
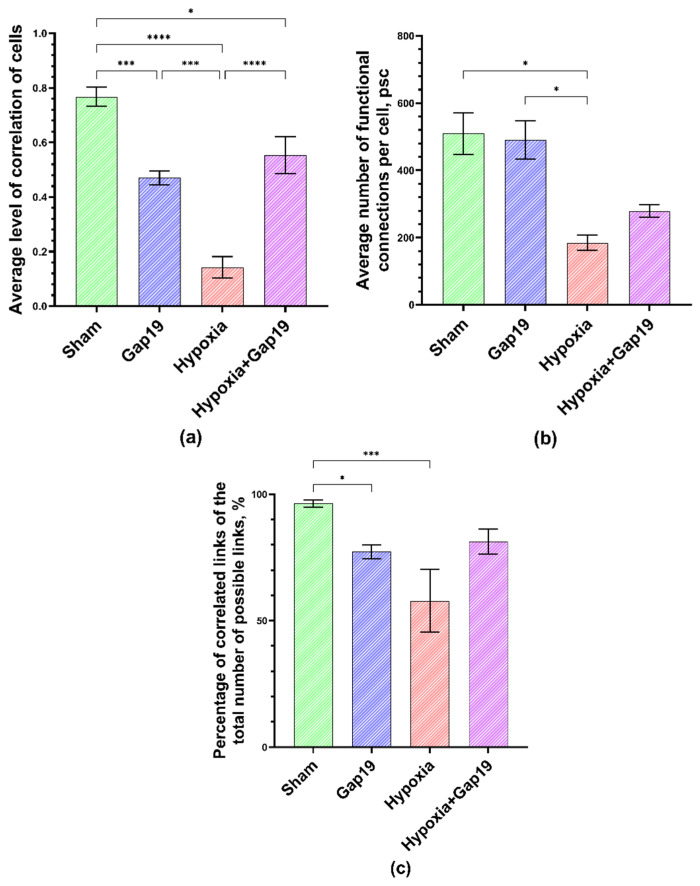
Main parameters of neuron–glial network activity in primary cortical cultures on the third day of the post-hypoxic period. (**a**) Mean correlation level of cells, (**b**) average number of functional connections per cell and (**c**) percentage of correlated connections from the total number of possible connections. * *p* < 0.05; *** *p* < 0.01, **** *p* < 0.001; Kruskal–Wallis test.

Therefore, the next stage of the study focused on the analysis of the main network characteristics of primary cortical cultures, paying particular attention to such parameters as the average level of correlation of Ca^2+^ oscillations, the average number of functional connections per cell and the percentage of existing correlated connections between cells from the total number of possible connections and the signal speed propagation (Figure 5).

The analysis showed that the neuron–glial networks in the “Sham” cultures had a high level of cell correlation (0.77 ± 0.03), with multiple functional connections in each cell on average (509.5 ± 62) and a high percentage of correlated connections from the mathematically calculated maximum possible number of connections (96.35 ± 1.41%) that is typical for this period of culture development in vitro [37,38].

Violation of astrocytic interactions through the blockade of Cx43 leads to a functional restructuring of the neuron–glial network. On the third day after applying the Gap19 blocker, the cultures showed a significant decrease in the level of correlation between cells in the network (0.47 ± 0.02) and in the percentage of existing correlated connections between cells (77.24 ± 2.78%) compared with the values of the “Sham” group.

Hypoxic damage leads to significant negative alterations in the functional architectonics of neuron–glial networks of primary cortical cultures. On the third day of the post-hypoxic period, in the “Hypoxia” group, there was a significant decrease in all analyzed parameters of network characteristics relative to the “Sham” group (Figure 5). This may indicate the death of functionally significant elements of neuron–glial networks, which increases the risk of impairments in synaptic transmission, which in turn can potentially result in the complete destruction of networks and, consequently, loss of brain function.

Blockade of Cx43 ameliorates the negative effect of hypoxic damage. In the “Hypoxia+Gap19” group, the level of cell correlation was comparable with the values of the “Gap19” group and was significantly higher than the values of the “Hypoxia” group (Figure 5). Despite a tendency toward a decrease in the average number of functional connections per cell (278.9 ± 18.72) and the percentage of existing correlated connections between cells (81.31 ± 4.9%), these indicators had no significant differences with the “Sham” group. Thus, the neuroprotective effect of Cx43 blockade was characterized not only by maintaining the main parameters of spontaneous calcium activity in primary cortical cultures but also contributing to the partial preservation of the functional architectonics of the neuron–glial network.

There were no statistically significant changes in the signal speed propagation between cells between the control and experimental groups (Figure S1 in Supplementary Materials).

**Figure 6:**
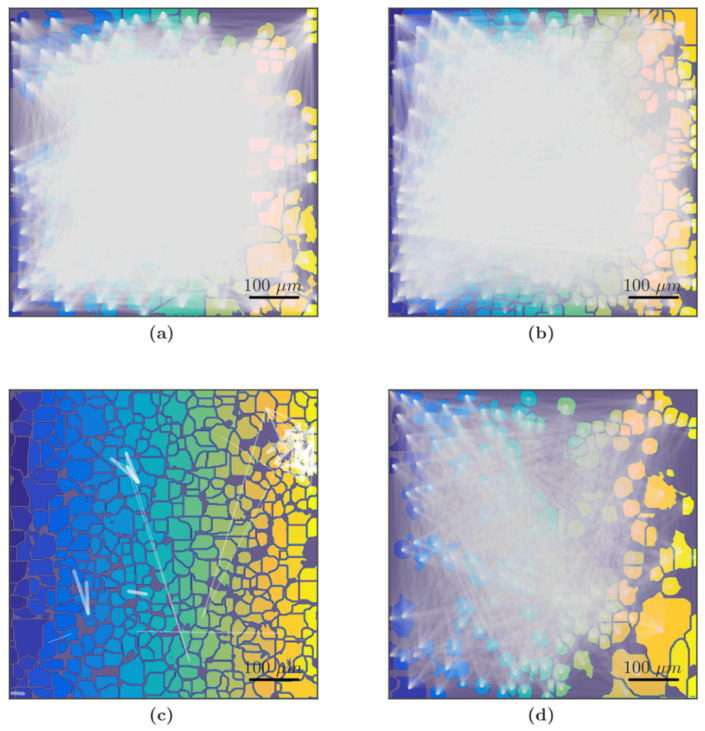
Representative correlation network graphs of primary cortical cultures on the third day of the post-hypoxic period. Each white line schematically represents the connection between cells. The line is plotted on the graph if the correlation level of Ca^2+^ oscillations is greater than the empirically calculated value 0.3, and the thicker the line, the higher correlation level. Colors of individual cells are chosen only for better recognition of cell boundaries: (**a**) Sham, (**b**) Gap19, (**c**) Hypoxia, (**d**) Hypoxia+Gap19. Scale bars: 100 μm.

The above-described changes in the network characteristics of the primary cortical cultures were confirmed by the analysis of correlation network graphs (Figure 6). Figure 6 shows that in the “Sham” and “Gap19” groups, the primary cortical cultures’ neuron–glial network had many functional connections between the cells that formed the neuron–glial network (Figure 6a,b).

In contrast, in the “Hypoxia” group, almost no cellular functional relationships suggested the degradation of the neuron–glial network (Figure 6c). This effect was not observed upon the blockade of Cx43. In the group “Hypoxia+Gap19”, the calcium signal communication in the preserved connections remained high (Figure 6d).

**Figure 7:**
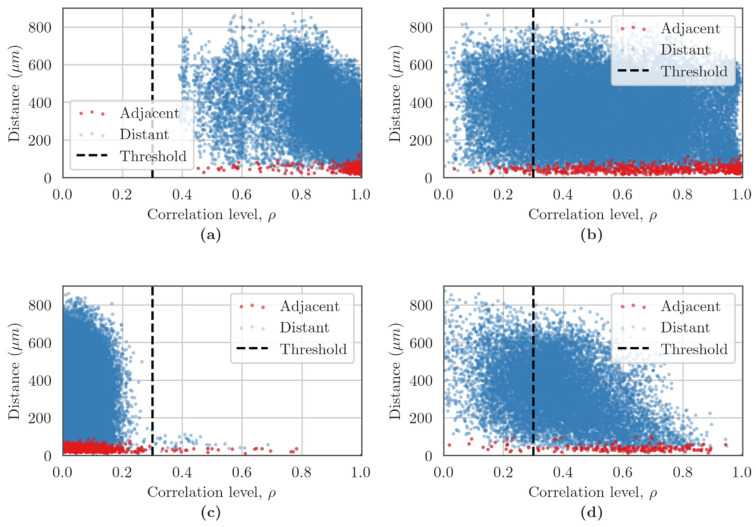
Dependence between the correlation level and the distance of cells pairs in primary cortical cultures on the third day of the post-hypoxic period. The adjacent cell pairs with their soma in direct contact are highlighted in red, and distant cell pairs in blue: (**a**) Sham, (**b**) Gap19, (**c**) Hypoxia, (**d**) Hypoxia+Gap19.

Analysis of the dependency of the distance between cells and the level of correlation of Ca^2+^ oscillations showed a high correlation of connections in the normal state between neighboring cells and cells without direct contact of soma (Figure 7). In the “Sham” group, most of the points reflecting the relationship between a pair of cells were concentrated in the area of maximum correlation from 0.8 to 1 (Figure 7a), which is consistent with previous studies [37]. Blockade of Cx43 under conditions of normoxia while maintaining a large number of functional relationships between the cells that form the neuron–glial network (Figure 6b) leads to a decrease in the level of correlation of cells, with the point cloud equally distributed from 0.1 to 1 (Figure 7b). Hypoxia leads to a dramatic decrease in the level of correlation between cells (Figure 7c). In the “Hypoxia” group, the point cloud was shifted to the beginning of coordinates, and the correlation level was in the range of 0 to 0.2, which is considered to indicate a lack of correlation. The blockade of Cx43 contributed to the partial preservation of the correlation relationships between the cells of the neuron–glial network during hypoxic damage; in the “Hypoxia+Gap19” group, the correlation level between a pair of cells equally ranged from 0.2 to 0.65.

**Figure 8:**
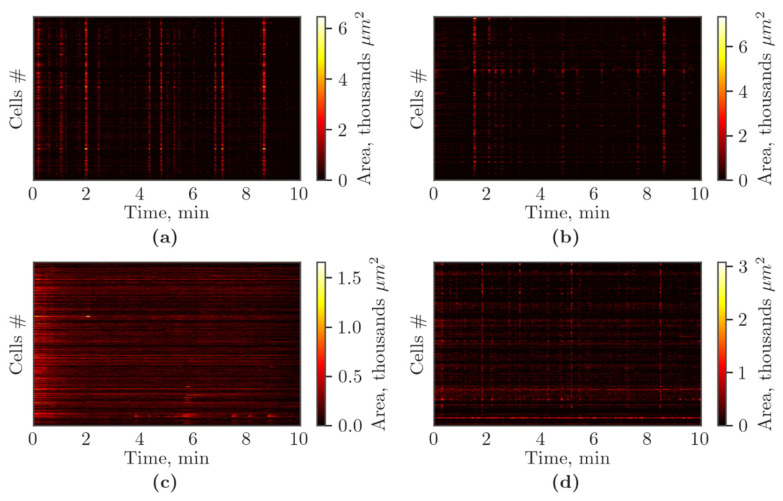
Representative raster diagrams of spontaneous calcium activity of primary cortical cultures on the third day of the post-hypoxic period. The *X*-axis represents the time of recording; the *Y*-axis represents the number (#) of active cells. The lighter the point, the higher area of fluorescence event in the cell: (**a**) Sham, (**b**) Gap19, (**c**) Hypoxia, (**d**) Hypoxia+Gap19.

Finally, we analyzed the profile of spontaneous Ca^2+^ oscillations in cells from primary cortical cultures using raster plots (Figure 8). The analysis revealed the presence of the synchronized activity of cells in the “Sham” and “Gap19” groups, accompanied by the appearance of superoscillations (Figure 8a,b). Hypoxia leads to significant alterations in the calcium activity profile, mainly represented by ungrouped single Ca^2+^ oscillations. The superoscillations were not detected in the “Hypoxia” group (Figure 8c). The blockade of Cx43 during hypoxic damage contributed to the partial preservation of the pattern of spontaneous Ca^2+^ activity of primary cortical cultures, mainly represented by synchronized Ca^2+^ oscillations. In the “Hypoxia+Gap19” group, superoscillations were also recorded but their duration (7.05 ± 2.61 s) was reduced compared to the “Sham” (14.85 ± 2.06 s) and “Gap19” (16.32 ± 3.28 s) groups (*p* < 0.05, Kruskal–Wallis test).

## 4. Discussion

For many years, hypoxia has continued to be among the key problems for global health. The versatility of the causes and development of the consequences of hypoxic damage challenges the timely diagnosis and selection of an effective therapeutic strategy. Research laboratories worldwide remain highly interested in discovering the specific molecular mechanisms of hypoxic damage and the features of the development of the body’s systemic responses and its adaptation to oxygen deficiency. Hypoxia affects almost all human organ systems, but its most detrimental effect is on the brain. The development of hypoxic-induced processes in the brain resulted in mitochondrial dysfunction [41,42], impaired synaptic transmission [43], activation of inflammatory [10] and apoptotic reactions [41], followed by nerve cell death and loss of brain function, which also aggravated the work of other organs and dramatically increased the risk of severe disability and often the death of the patient.

For a long time, the pathogenetic mechanisms of hypoxia were considered at the level of single neurons [44]. Only in the last decade, the attention of researchers was switched to the study of neural networks as the main functional units of the CNS responsible for the implementation of processing, storage and transmission of information, as well as for the implementation of higher cognitive functions, including memory, consciousness and emotional reactions [45]. This approach, using modern methods of neurobiology, makes it possible to analyze the tiny mechanisms of restructuring of the functional architectonics of neural networks under hypoxic damage, as well as the contribution of each element of the network to the development of the functional network response to the stress factor [46,47].

However, accumulating evidence indicates that physiological and pathological neural network processes are critically dependent on the activity of glial cells, particularly astrocytes. By forming their networks, astroglial cells perform not only homeostatic and trophic functions for neurons [48], but also, more importantly, being in a close mutual relationship with neural networks, they can regulate their functional activity, thereby being active participants in signal transmission and having an impact on brain functioning, including cognitive and information processing [15,48,49,50].

Therefore, to fully understand the pathological processes of various origins in the CNS, including hypoxia, and to develop new therapeutic methods, it is urgently necessary to study the functional activity of complex neuron–glial networks. In this context, primary cultures of brain cells can serve as a useful biological model for studying the morphological and functional features of neuron–glial networks; the rationality of using the model in such in vitro studies has been proven previously [33,34,35,51]. Herein, using primary cortical cultures, we investigated the role of inter-astrocytic interactions in the functional adaptation of neuron–glial networks in the early and late periods after acute normobaric hypoxia modeling.

Our attention was focused on the key links of gap junctions that form astrocytic networks, particularly Cx43, which, according to the literature, may be critically involved in promoting hypoxia–ischemia injury [21,52].

Hypoxic damage primarily leads to dysfunction of the glutamatergic system; dysregulation of glutamate leads to its accumulation in the extracellular space and a simultaneous increase in the concentration of intracellular Ca^2+^, which, as a result of the subsequent activation of a number of biochemical reactions, determines the death of nerve cells [53]. This is why we analyzed the levels of expression of metabotropic glutamate mGluR2 and mGluR5 receptors. In normal conditions, mGluR2 and mGluR5 receptors play a significant role in regulating the glutamatergic system and synaptic plasticity and determine the resistance of both neurons and glial cells to excitotoxic influences [54]. Activation of mGluR2 receptors leads to the appearance of inhibitory effects due to a decrease in intracellular cAMP or the activity of voltage-dependent Ca^2+^-channels [55,56]. An increase in mGluR5 expression, in turn, can lead to the activation of synaptic transmission or an increase in the expression of synaptic plasticity genes, and may have a neuroprotective effect [57,58].

It is worth emphasizing that the blockade of Cx43 under normoxic conditions leads to an increase in the expression of both mGluR2 and mGluR5 receptors, which could have a compensatory effect, resulting in the long-term modulation of spontaneous calcium activity in primary cortical cultures. Despite the persistence of the Ca^2+^ activity profile typical for primary neuronal cells of this cultivation period [35,37], cultures of the “Gap19” group tended to decrease in the number of functionally active cells. Violation of astrocytic interactions led to the restructuring of the functional architectonics of neuron–glial networks, characterized by a decrease in the level of correlation between network cells and the percentage of existing correlated connections between cells in the presence of a large number of functional connections. Thus, it is fair to conclude that astrocytic networks are important elements of neuron–glial networks, providing a high level of correlated connection between network cells and, consequently, a consolidated network response.

In hypoxic damage, the pronounced increase in the expression of mGluR2 and mGluR5 receptors observed by us in the early post-hypoxic period (day 1) can be regarded as the activation of adaptive processes aimed at maintaining a balance between inhibitory and excitatory processes in nerve cells and preventing the development of excitotoxicity [59]. However, the remaining high level of expression of mGluR5 at a later post-hypoxic period (day 3) can lead to the launch of intracellular signaling systems that activate the release of Ca^2+^ ions from the endoplasmic reticulum storage and increase the concentration of intracellular free Ca^2+^, followed by the development of excitotoxicity and cell death [60]. We observed this while studying the spontaneous calcium activity of primary cortical cultures. On the third day of the post-hypoxic period, in the “Hypoxia” group, a dramatic decrease in the percentage of active cells and a change in the profile of calcium activity formed by disparate single Ca^2+^ oscillations were found. In addition, hypoxia leads to significant negative alterations in the functional architectonics of neuron–glial networks, resulting in a decrease in all studied network characteristics. In cultures, there were almost no functional correlated relationships between cells, suggesting the death of functionally significant connections of neuron–glial networks and the degradation of the neuron–glial networks.

The blockade of Cx43 under hypoxic injury has a pronounced neuroprotective effect. On the first day of the post-hypoxic period, an increased level of expression of mGluR2 and mGluR5 receptors was established; however, after two days, the level of mGluR2 returned to the level of intact values. Since mGluR5 is widely present on astrocytes, while mGluR2 expression prevails on neuronal cells [61,62,63], it can be assumed that under hypoxic conditions, even with the blockade of inter-astrocytic interactions, increased expression of mGluR5 is aimed to increase glutamate uptake and prevent the hyperexcitability of neurons, thereby preventing the development of excitotoxicity. When uncoupling the consolidated response of the astrocytic network, the absence of a significant increase in mGluR2 expression suggests the triggering of alternative molecular mechanisms of cell adaptation to hypoxic damage, which require further in-depth study. In addition to the regulation of glutamate release [25], one of the possible molecular mechanisms of the neuroprotective action of the blockade of astrocytic Cx43 hemichannels by Gap19 under hypoxia–ischemia conditions can be realized through the activation of the JAK2/STAT3 signaling pathway that mediates the pronounced anti-apoptotic effect [24], which contributes to the maintenance of cell viability and functional activity. Thus, we showed that the blockade of Cx43 ameliorated the negative effect of hypoxic damage on the spontaneous calcium activity of cells in primary cortical cultures. The observed neuroprotective effect was expressed not only in the maintenance of the main parameters of the spontaneous calcium activity of primary cortical cultures at the physiological level, but also contributed to the partial preservation of the profile of Ca^2+^ oscillations with synchronized network events—superoscillations. In addition, the blockade of Cx43 contributed to the maintenance of the functional architectonics of the neuron–glial network, with the preservation of calcium signal communications between cells at a highly correlated level. Thus, our results demonstrate the crucial importance of astrocytic networks in functional brain adaptation to hypoxic damage and may be a promising target for the development of rational anti-hypoxic therapy. Considering the results obtained in the present study, an equally interesting question regards the peculiarities of signal propagation between neural networks that will be observed when inter-astrocyte interactions are disconnected in a normal state and under hypoxic damage. Indeed, this is an intriguing direction for our upcoming research; more detailed experiments on the spontaneous bioelectrical activity of neural networks in primary brain cell cultures using multielectrode array technology and original mathematical data analysis algorithms are expected.

## 5. Conclusions

Under normoxia, the blockade of astrocytic Cx43 leads to an increase in the expression of metabotropic glutamate receptors mGluR2 and mGluR5, as well as the prolonged modulation of spontaneous calcium activity in primary cerebral cortex cell cultures, primarily expressed in the restructuring of the functional architectonics of neuron–glial networks by reducing the levels of correlation between network cells and the percentage of existing correlated connections between cells. The blockade of Cx43 during hypoxic injury has a pronounced neuroprotective effect. In the late post-hypoxic period, against the background of the persistently increased expression of mGluR5 receptors, a decrease in mGluR2 expression to a physiological level was found; it suggests the launch of alternative molecular mechanisms for cell adaptation to hypoxic damage. In addition, the blockade of Cx43 contributed to the maintenance of both the main parameters of the spontaneous calcium activity of primary cultures and the functional architectonics of the neuron–glial network, while maintaining the profile of Ca^2+^ oscillations and Ca^2+^ signal communications between cells at a highly correlated level. The results of our study demonstrate the crucial importance of astrocytic networks in functional brain adaptation to hypoxic damage and may be a promising target for the development of rational anti-hypoxic therapy.

## Data Availability

The data used to support the findings of this study are available from the corresponding author upon request.

## Supplementary Materials

The following supporting information can be downloaded at: https://www.mdpi.com/article/10.3390/membranes12100948/s1, Figure S1: Signal speed propagation between cells in neuron–glial networks of primary cortical cultures on the third day of the post-hypoxic period; Videos S1–S4: Representative recordings of spontaneous calcium activity of primary cortical cultures on the third day of the post-hypoxic period: (S1) Sham, (S2) Gap19, (S3) Hypoxia, (S4) Hypoxia+Gap19. Each video file compresses an original 10-min recording of spontaneous calcium activity of the primary cortical culture with the registration rate of two frames per second.
