# Supplementary figures and images for: Unravelling Contributions of Astrocytic Connexin 43 to the Functional Activity of Brain Neuron–Glial Networks under Hypoxic State In Vitro
Source: Membranes (Basel). 2022 Sep 28;12(10):948. doi: 10.3390/membranes12100948 (PMC9609249; doi:10.3390/membranes12100948)

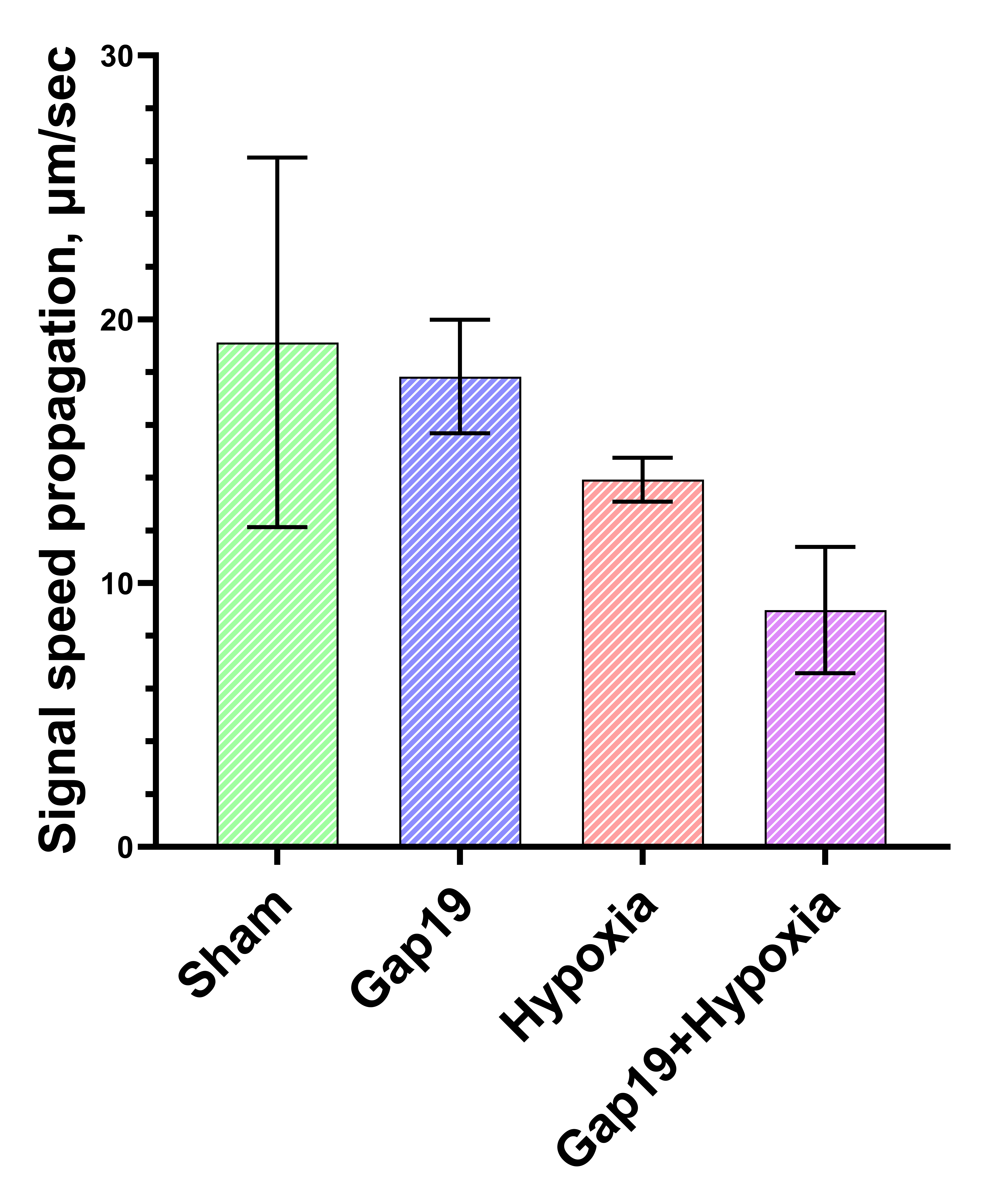

Supplement: Supplementary file 1 [file membranes-12-00948-s001.zip › Figure S1.tif]
